# Co-Designing a Digital App to Support Young People’s Patient and Public Involvement and Engagement (VoiceIn): Development and Usability Study

**DOI:** 10.2196/53394

**Published:** 2024-10-24

**Authors:** Alison Branitsky, Penny Bee, Sandra Bucci, Karina Lovell, Simon Foster, Pauline Whelan

**Affiliations:** 1 Division of Psychology and Mental Health, School of Health Sciences, Faculty of Biology Medicine and Health, Manchester Academic Health Science, The University of Manchester, Oxford Road, Manchester, M13 9PL, United Kingdom; 2 Complex Trauma and Resilience Research Unit, Greater Manchester Mental Health NHS Foundation Trust, Manchester, United Kingdom; 3 Psychosis Research Unit, Greater Manchester Mental Health NHS Foundation Trust, Manchester, United Kingdom; 4 Division of Nursing, Midwifery and Social Work, School of Health Sciences, Faculty of Biology, Medicine and Health, Manchester Academic Health Science, The University of Manchester, Manchester, United Kingdom; 5 NIHR Applied Research Collaborative - Greater Manchester, Manchester, United Kingdom; 6 Division of Informatics, Imaging & Data Sciences, School of Health Sciences, Faculty of Biology, Medicine and Health, Manchester Academic Health Science, The University of Manchester, Oxford Road, Manchester, M13 9PL, United Kingdom, 44 0161 275 5096; 7 GM.Digital Research Unit, Greater Manchester Mental Health NHS Foundation Trust, Manchester, United Kingdom

**Keywords:** patient and public involvement and engagement, PPIE, digital mental health, young people, co-design, mental health

## Abstract

**Background:**

While patient and public involvement and engagement (PPIE) is now seen as a cornerstone of mental health research, young people’s involvement in PPIE faces limitations. Work and school demands and more limited independence can make it challenging for young people to engage with PPIE. Lack of ability or desire to attend face-to-face meetings or group discussions can further compound this difficulty. The VoiceIn app and digital platform were codeveloped by a multidisciplinary team of young people, mental health researchers, and software designers, and enables young people to engage directly with PPIE opportunities via a mobile app.

**Objective:**

This paper aims to describe how VoiceIn was developed through a series of co-design workshops with relevant stakeholders, specifically (1) how the initial design of VoiceIn was informed and driven by focus groups with young people, mental health professionals, and PPIE leads; (2) how VoiceIn was refined through collaboration with the aforementioned stakeholders; (3) the priorities for an app to support PPIE; (4) the key features necessary in the PPIE app; and (5) the recommended next steps in testing and deploying the digital platform.

**Methods:**

Initial co-design workshops took place with young people, mental health professionals, and PPIE leads to identify key features of an app to support PPIE. A series of VoiceIn design prototypes were developed and iterated based on the priorities and preferences of the stakeholders. The MoSCoW (must have, should have, could have, won’t have) prioritization method was used throughout the process to identify priorities across the different stakeholder groups.

**Results:**

Co-design with young people, mental health professionals, and PPIE leads supported the successful development and improvement of the VoiceIn app. As a result of this process, key features were identified, including allowing for various modes of providing feedback (eg, polls and comments), reviewing project updates, and expressing interest in categories of research. The researcher platform was developed to support multimedia uploads for project descriptions; a jargon detector; a dedicated section for providing project updates; and a visually appealing, user-friendly design. While all stakeholder groups emphasized the importance of allowing app users to engage with the app in various ways and for there to be ongoing progress updates, group differences were also noticed. Young people expressed a desire for incentives and rewards for engaging with the app (eg, to post on their public social media profiles), and mental health professionals and PPIE leads prioritized flexibility in describing the project and its PPIE needs.

**Conclusions:**

A co-design approach was pivotal to the development of the VoiceIn app. This collaborative approach enabled the app to meet the divergent needs of young people, mental health professionals, and PPIE leads. This process mirrored the aspirations of PPIE initiatives by cocreating a digital health research tool with key stakeholders.

## Introduction

Patient and public involvement and engagement (PPIE) has become a cornerstone of best practice in mental health research. PPIE is premised on the notion that research should be carried out “by” or “with” those who the research is intended to benefit, rather than “to” or “for” them [1]. At its core, PPIE values the primacy of subjective lived experience in knowledge construction and emphasizes a way of producing science where experts by experience are active co-designers and co-researchers through the entire research process [2,3]. PPIE provides valuable opportunities for addressing the democratic deficit and power imbalance that exist in much of health research by giving an equal voice to the intended beneficiaries of such research [4].

PPIE has numerous demonstrable benefits, including increasing the impact of research by homing in on the questions most relevant to stakeholders, designing more appropriate and targeted methodologies, addressing ethical tensions that exist between various stakeholder groups, and exploring novel ideas that may not have been generated by researchers alone [5-10]. By rooting findings and reports in user experience, outcomes are likely to be more relevant, relatable, and understandable to the public, thus enhancing both dissemination and implementation [6,7].

Young people can be valuable and engaged research partners. However, recent scoping research has demonstrated that young people’s expertise may be underused. A 2018 United Kingdom–based survey of public contributors to the National Institute for Health and Social Care Research found that only 2% of those involved were younger than 25 years [11]. Similarly, Rouncefield-Swales et al [12] found that where young people were involved in PPIE, their level of involvement was varied, the impact of their involvement was often unknown, and details of precisely how PPIE contributions were integrated into projects were lacking in written reports. However, in regions where a specific effort was made to include young people in PPIE, their involvement surpassed those of any other age group [13]. Given that the principle of young people’s involvement is clearly enshrined in international policy [14,15], there is a strong impetus to further develop ways in which young people can meaningfully engage with all stages of health research.

Involving young people in traditional, face-to-face PPIE can prove challenging. School and work commitments, limitations on independence and flexibility, resource and financial constraints, and rapid developmental and lifestyle changes can make it difficult for young people to engage with PPIE long-term [12,16,17]. Furthermore, meeting face-to-face with various stakeholders is not a suitable environment for all young people to feel comfortable and empowered to contribute. Consequently, researchers often struggle to recruit [18] and retain [19] young people to be involved in PPIE. Research projects may be further limited by recruiting young people from small geographic areas, thereby limiting the generalizability of research input and findings [20]. As a result, there is a clear need to re-evaluate the current adult-centric modes of PPIE participation and spearhead innovative means of youth participation [21,22].

Shimmin et al [23] argue that many current PPIE practices are limited in their recognition of the real complexities of people’s lives. As a result, many individuals who carry the greatest burden of illness, particularly those with marginalized or excluded social identities and those experiencing various forms of systemic oppression, are less likely to have their voices heard in the sphere of PPIE. When looking at PPIE through the lens of health equity, there is a strong impetus to maximize diversity both in terms of social identity and in modes of meaningful participation.

Digital health technology provides a promising avenue for complementing existing PPIE methods by expanding participation opportunities to an audience that may not otherwise be reached. To our knowledge, there is currently no dedicated digital platform to provide PPIE opportunities to young people in real-time. To facilitate young people’s involvement in mental health research and to make PPIE opportunities more accessible and inclusive, we built the “VoiceIn” PPIE digital platform, which we introduce here. VoiceIn acts as a 2-way PPIE toolkit whereby researchers can solicit PPIE input from young people, and young people provide feedback in their own time via a mobile app.

VoiceIn is a digital platform aimed at enabling young people to contribute easily and quickly to PPIE activities. It particularly aims to support young people who may ordinarily be unable or unwilling to attend traditional, face-to-face PPIE groups or activities. VoiceIn has been specifically designed to be user-friendly, fun, and easy to fit into young people’s lifestyles. The app allows young people to give feedback on projects that align with their interests or lived experiences. Researchers can ask for specific input from PPIE participants on various aspects of the project, from the initial shaping of research topics and design to methodological feedback. Participants can provide feedback in the form of polls or free text. VoiceIn emphasizes ongoing collaboration between participants and researchers: researchers provide frequent updates to highlight the impact of PPIE on project development, and participants have the ability to track their contributions across various projects.

VoiceIn was co-designed with young people, mental health researchers, and leaders of community-based PPIE groups. The purpose of this paper is to detail (1) how the initial design of VoiceIn was informed and driven by focus groups with young people, mental health professionals, and PPIE leads; (2) how VoiceIn was refined through collaboration with the aforementioned stakeholders; (3) the central priorities for an app to support PPIE; (4) the key features necessary in the PPIE app; and (5) the recommended next steps in testing the digital platform.

## Methods

### Study Setting, Participants, and Recruitment

A convenience sampling approach was adopted for the phase 1 workshops, with participants recruited from established local young people’s advisory groups and through team networks. The phase 1 co-design workshops were run as exploratory patient and public involvement activities with locally established young people’s advisory groups, mental health staff, and PPIE leads. Convenience sampling was adopted at this stage to assess initial interest in the idea of the VoiceIn app and to determine high-level requirements. For the phase 2 co-design workshops, a purposive sampling approach was adopted. For phase 2, recruitment targeted individuals outside of immediate research team networks and aimed to reach people who were not previously involved in the project. The inclusion criterion for young people’s groups was a requirement to be older than 16 years with the capacity to consent. The inclusion criterion for mental health professionals was a requirement to be adult staff working in mental health services for children or young people. For PPIE leads, the inclusion criterion was to have a role in running and coordinating PPIE activities for young people in a public sector or voluntary organization. Input from young people was seen as key to the development of a usable and engaging app, and for phase 2, we sought to increase the number of young people involved in the workshops (from 4 to 8). Around 8 people were deemed the maximum number we could support through a web-based workshop and sufficient for capturing ideas to progress the development of the app. Mental health professionals and PPIE leads for phase 2 were approached through national networks for mental health and PPIE. National organizations and third-sector or charitable organizations were approached by email at this stage.

### Co-Design Workshops

#### Overview

**Figure 1.**
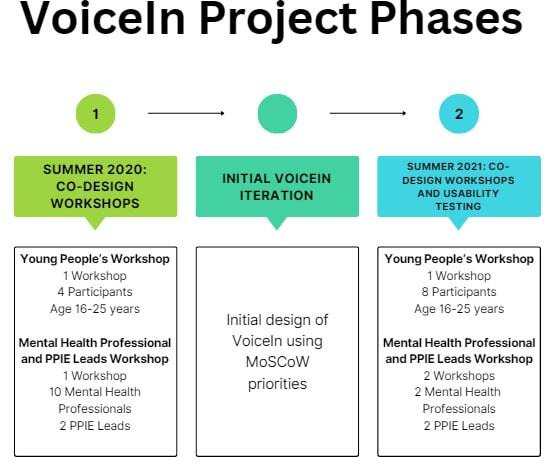
Co-design workshops timeline. MoSCoW: must have, should have, could have, won’t have; PPIE: patient and public involvement and engagement.

Between 2020 and 2021, a series of co-design workshops were conducted with young people, mental health professionals, and PPIE leads. An overview of the timeline of the workshops and their participants is outlined in Figure 1.

**Figure 2.**
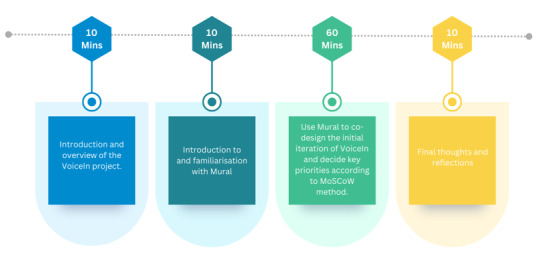
Structure of phase 1 co-design workshops. MoSCoW: must have, should have, could have, won’t have.

Workshops were held on Zoom and lasted 90 minutes. All workshops followed a similar structure: participants and the research team introduced themselves, participants were provided with an overview of the VoiceIn project, and the rest of the meeting was dedicated to exploring specific topics related to app co-design. The specific workshop structure is provided in Figures2  3. For larger group sizes, participants were assigned to breakout rooms with 2 to 3 people and 1 member of the research team. For smaller groups, a single group was maintained throughout. Topics of discussion were decided a priori and are described in Figure 4. Time was also allocated to allow for discussions of topics that arose organically during the meeting. The software collaboration tool Mural [24] was used in the young people’s workshop to enable participants to share their thoughts on a collaborative whiteboard.

**Figure 3.**
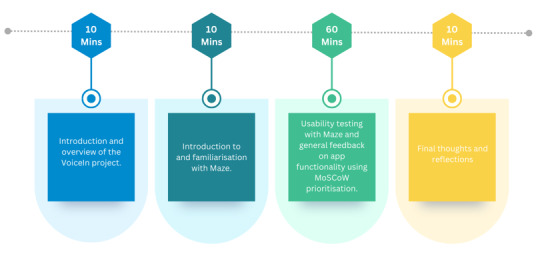
Structure of phase 2 co-design workshops. MoSCoW: must have, should have, could have, won’t have

**Figure 4.**
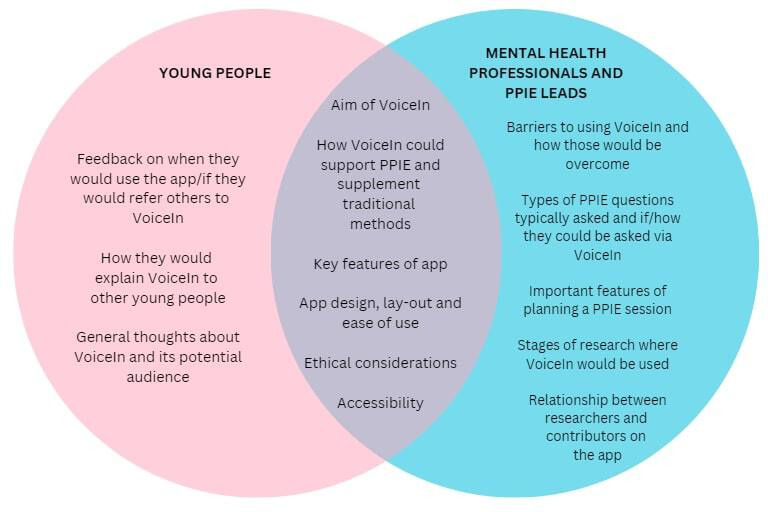
Topics discussed in co-design workshops. PPIE: patient and public involvement and engagement.

#### MoSCoW Prioritization

To enable the identification of priorities for the app, the MoSCoW (must have, should have, could have, won’t have) prioritization method was used [25]. This method is an oft-used method in software project management to determine the most crucial deliverables across groups of stakeholders. In the MoSCoW approach, app features were ranked by each stakeholder group as “must have,” “should have,” “could have,” and “won’t have.” “Must have” items were considered nonnegotiable by stakeholders; “should have” items were those considered to have significant value but would not affect the key functionality of the app; “could have” items were additional desirable features that were not necessarily related to the core goals of VoiceIn, while items classified as “won’t have” were not priorities for the current VoiceIn release but may be considered for implementation in the future. Focus group facilitators supported discussion when different priorities were identified, aiming to arrive at a consensus decision. Where this was not possible, the majority of contributed opinions were used to determine the priority level assigned to each feature.

#### Agile Software Development

The software team adopted an Agile approach to software development, building the software iteratively and refining the platform based on feedback at the workshops. Following each co-design workshop, the software engineering team discussed the technical feasibility of identified requirements; some items were put on hold for implementation due to the complexity of the implementation involved and the time or budget constraints of the project. Software engineers then worked to incorporate the features ranked “must have” and “should have” in the MoSCoW process into the next iteration of the app for presentation at the subsequent workshop.

#### App Technical Development

The VoiceIn app and digital platform were developed by the specialist Digital Health Software and Platforms team based at the University of Manchester [26]. The software team has expertise in developing digital health research technology. An Agile software development approach was adopted with the software team delivering the digital platform iteratively across a number of Agile sprint cycles. A hybrid app framework (Capacitor by Ionic) was used for ease of deployment to multiple devices and to align with expertise within the software team [27]. The web back end was supported by an open-source relational database (MySQL) [28] with a lightweight open-source framework Vue.js used for the front end [29].

#### Usability Tasks

In the phase 2 co-design workshops, participants were asked to complete a usability testing exercise on a platform called Maze [30]. Maze is a commercial product development platform that enables usability and prototype testing of new products, including web and mobile apps. Participants used their own devices to access Maze; Maze was accessible with all Android and iOS devices. In the young people’s workshops, participants were asked to provide project feedback on a prototype of the app. In the mental health professionals and PPIE leads’ workshops, participants were asked to create a mock project. After completing their respective task, participants were asked to rate the difficulty of the assignment and provide feedback about what, if anything, they found difficult and if anything was missing.

### Ethical Considerations

Informed consent was obtained from all workshop participants. Only participants older than 16 years with the capacity to consent were included in the workshops. Ethical approval for the phase 2 co-design workshops was granted by the University of Manchester Research Ethics Committee (REF 2021-10864-17849). Ground rules were established by the workshop facilitators at the start of each workshop to clarify the workshop’s purpose and guide the conduct of the workshop.

## Results

### Phase 1 Co-Design Workshops: Young People

Preliminary co-design workshops with young people demonstrated a diverse array of preferences for engaging with the VoiceIn app. There was a general consensus that young people preferred to be involved throughout the entire research process, stating that PPIE should be the “driving force” behind research projects. Some participants felt it would be particularly interesting and rewarding to be involved in the design of research projects and identification of key research questions and priorities; others preferred more discrete and concrete tasks such as providing specific feedback on topic guides, picking questionnaires, and coproducing the terms of reference for ongoing PPIE support during projects. Participants highlighted the need for the app to fit into the day-to-day lives of young people, noting that quick responses such as polls and surveys should be included since some young people will not have the time or ability to review lengthy documents and give detailed feedback. Young people also suggested including rewards and incentives to encourage engagement with the app, considering ways for young people to engage with one another within the app, and including sections such as “meet the researcher” to make projects more contextualized and relational.

Young people identified a desire and need for training on research processes generally when long-term involvement PPIE involvement was necessary. Although this feedback fell outside the scope of the app (which was primarily intended to capture “quick” feedback on early-stage research ideas or short responses to specific questions during the lifetime of a project), it was noted that a training module within the app describing the research life cycle and processes would be a useful addition to consider for future implementation. Additional training material (eg, a research handbook) has been developed as a result of these conversations, but likewise falls beyond the scope of what could be included in the app.

### Phase 1 Co-Design Workshops: Mental Health Researchers and PPIE Leads

Similar to the young people, mental health researchers and PPIE leads aimed to seek ongoing PPIE input throughout the research process. In particular, they predicted using the app to explore possible areas of research and define research questions, seek comments on recruitment methods and data collection tools, and disseminate results through the co-development of communication plans. Researchers noted, however, that they would not use the app to seek PPIE input for determining specific outcome measures or data analysis, due to the specialist knowledge required for both tasks.

### MoSCoW Prioritization and Initial VoiceIn Iteration

#### Overview

During both the phase 1 and phase 2 co-design workshops, participants were asked to rate different app features according to the MoSCoW prioritization method in order to determine their priority. Every effort was made to ensure that items ranked as “must have” and “should have” were included in the initial iteration of VoiceIn. Some items were deemed outside of scope for this version (due to time or budget limitations) but were noted for future iterations of the platform.

#### General Capabilities

A summary of stakeholders’ priorities regarding the general capabilities of VoiceIn is described in Table 1. Across stakeholder groups, providing project progress updates was identified as a clear priority; as such, VoiceIn was built to prompt mental health researchers to provide relevant updates, and a dedicated “project updates” section was built into the mobile app. Stakeholders likewise noted both global (eg, technical support section, terms of reference) and project-specific (eg, having the ability to decide on research questions and topics) as key priorities.

**Table 1.** MoSCoW^a^ general capabilities priorities for user-facing mobile app interface capabilities.

Description of general capabilities	Group				
	Young people	Mental health researchers	PPIE^b^ leads	Included?	Reason for exclusion
Input to decide on research topics or questions	Must Have	Must Have	Should Have	Yes	—^c^
Information on the role of PPIE in shaping the research	Must Have	Must Have	—	Yes	—
Project progress updates	Must Have	Must Have	Must Have	Yes	—
Technical support section	Must Have	Must Have	—	Yes	—
Notifications for new research opportunities	Must Have	—	—	Yes	—
Ability to collect research data within the app	Must Have	—	Should Have	No	As the app is specifically to aid with PPIE, rather than carry out research procedures, this falls beyond the scope of the app
Contributor leader board to demonstrate users who have provided the most contributions	Must Have	Could Have	—	No	Leader board ultimately excluded to demonstrate that all contributions are valuable, regardless of the number of contributions per user
Credit or time-based rewards for contributions	Must Have	Must Have	—	No	Complexity of implementation and variety of reward types discussed precluded the provision of rewards in the initial version. Further discussion is required to scope the requirements, planned for the next phase
Recognition for user inputs	—	Should Have	—	No	PPIE contributions recognized on a project-by-project basis and therefore fall beyond the scope of the app
Terms of reference or expectations	—	Must Have	Must Have	Yes	—
Data access or GDPR^d^ information	—	Must Have	—	Yes	—
Users to assist with publicizing study or helping with recruitment	Should Have	Must Have	Should Have	No	Formal recruitment activities are outside the current PPIE scope of VoiceIn but project information and contact details can be provided
Glossary of research terminology	—	Must Have	—	No	Resource limitations
Notice that content may cause distress	—	Must Have	—	Yes	—
Project categories are lay-friendly and not diagnosis-based	—	Must Have	—	Yes	—
Notifications for researcher to put project updates on the app	—	Must Have	—	Yes	Researchers are reminded on login to the website
Payment policies	—	—	Must Have	No	This is actively under consideration of how to recognize contributions fairly and safely within the app
Contributions exportable to LinkedIn or CV^e^	Should Have	—	Must Have	No	Technical constraints limit the ability to create exportable contributions at this time. But under consideration for future
Reminders for research milestones and dates for feedback to be returned	Should Have	—	—	Yes	—
Ideas lab to generate novel research ideas	—	Should Have	—	No	Resource limitations, but under consideration for future iteration
Demonstrate impact of research projects	—	Should Have	Should Have	Yes	—
Create a database of people interested in research	—	—	Should Have	No	Data protection limitations prohibit the creation of this type of database
Hear about new funding and related work	Could Have	—	—	Yes	—
Certificates of involvement	Could Have	Could Have	—	No	Technical constraints prevent the creation of certificates within the app at this time
Questions to assess users’ mood and offer support	—	Could Have	—	No	A support section is included in the app, but ongoing monitoring of users’ moods is beyond the scope of the app and may not be acceptable for some users
Debrief area	—	Could Have	—	Yes	—
Area to share lessons learned	—	—	Could Have	No	Social features between researchers are not currently enabled
Translation into other languages	—	—	Could Have	No	Current limitations on project resources prevent translating the app into other languages

a MoSCoW: must have, should have, could have, won’t have.

b PPIE: patient and public involvement and engagement.

c Not applicable.

d GDPR: General Data Protection Regulation.

e CV: curriculum vitae.

#### Project Details

There was no clear consensus across stakeholder groups on the priorities pertaining to study details; however, sharing information about funding, including the project dates, and having the ability to upload documents were all identified as priorities among mental health researchers. Project detail priorities across stakeholder groups are outlined in Table 2.

**Table 2.** MoSCoW^a^ project details priorities for user-facing mobile app interface capabilities.

Description of project details	Young people	Mental health researchers	PPIE^b^ leads	Included?	Reason for exclusion
Information about funding	Could Have	Must Have	—^c^	Yes	—
Study date ranges as part of the project details	—	Must Have	—	Yes	—
Ability to upload documents	—	Must Have	Should Have	Yes	—
Addition of images/video/graphics to project description	—	Should Have	—	Yes	—
Researcher profile	—	—	Should Have	Yes	—
Flexible options for entering project details	—	Could Have	—	Yes	—
Addition of inclusion or exclusion criteria	—	Won’t Have	—	No	Inclusion or exclusion criteria can be included as free text; there will not be a separate section to input these criteria. Participant recruitment is not the goal of the app

a MoSCoW: must have, should have, could have, won’t have.

b PPIE: patient and public involvement and engagement.

c Not applicable.

#### Project Feedback

Stakeholders agreed that it was vital for app users to provide quick feedback via polls and likewise indicated that having the ability to give feedback on both participant and staff-facing material was a key priority. Project details priorities across stakeholders are outlined in Table 3.

**Table 3.** MoSCoW^a^ project feedback priorities for user-facing mobile app interface capabilities.

Description of project feedback	Young people	Mental health researchers	PPIE^b^ leads	Included?	Reason for exclusion
Ability to provide feedback via polls	Must Have	Must Have	—^c^	Yes	—
Sharing project materials (eg, PIS^d^, consent forms)	Must Have	—	—	Yes	This is possible but sharing lengthy documents via the app is discouraged as this is not the goal of the app
Providing feedback on project materials (eg, PIS, consent forms)	Must Have	—	Should Have	Yes	—
Method to select data collection tools	Should Have	—	Should Have	Yes	—
Area to review advertisements for staff, steering groups, etc	Should Have	—	Should Have	Yes	—
Area to analyze and interpret data	Should Have	—	—	Yes	—
Help provide feedback on qualitative interview questions	Should Have	—	—	Yes	—
Ability for users to pause and come back later to contribute	—	Should Have	—	—	—
Help decide who may be interested in hearing about the results	Could Have	—	—	Yes	—
Ways for contributors to share ideas (eg, recruitment, dissemination)	—	Could Have	—	Yes	—
Reviewing previous research	Won’t Have	—	—	No	Not desired by young people

a MoSCoW: must have, should have, could have, won’t have.

b PPIE: patient and public involvement and engagement.

c Not applicable.

d PIS: participant information sheet.

#### Training and External Links

Participants across stakeholder groups indicated a desire for the app to have the capability to both provide project-specific training and provide links to relevant resources and opportunities beyond the app. Resource limitations preclude maintaining a comprehensive list of external training or providing internal training via the app. However, links to additional opportunities and resources can be provided on a project-specific level via the project updates page. Training and external links priorities across stakeholder groups are outlined in Table 4.

**Table 4.** MoSCoW^a^ training and external links priorities for user-facing mobile app interface capabilities.

Description of training and external links	Young people	Mental health researchers	PPIE^b^ leads	Included?	Reason for exclusion
Project-specific training	Must Have	—^c^	—	No	However, contextual background project information can be provided
Tailored training for participants and researchers	Should Have	—	Should Have	No	—
Hear about opportunities for future training or learning	Should Have	—	—	Yes	Can be supported per project updates
Job opportunities and information about other ways to be involved in research	Should Have	—	Could Have	Yes	Can be supported per project updates
Advertise internal or external development opportunities	Could Have	—	—	Yes	Can be supported per project updates
Training on research methodology	Won’t Have	—	—	No	—
Information on meeting with peers and community groups	—	—	Should Have	No	—
Links to wider PPIE networks and opportunities	—	Could Have	Could Have	No	Planned for future iteration
Expand app to other audiences or ages	—	—	Must Have	Yes	—
Stakeholder consensus meeting	Won’t Have	—	Must Have	No	—

a MoSCoW: must have, should have, could have, won’t have.

b PPIE: patient and public involvement and engagement.

c Not applicable.

#### Social Features

The various stakeholder groups identified different priorities for social features within VoiceIn, with the “ability for users to connect with one another, the public and other researchers” being rated as the top priority. While resource limitations preclude the ongoing monitoring necessary to enable social features, mental health researchers are encouraged to include information about the research team on the project details page, and provide ongoing updates to foster engagement with app users. Social features priorities across stakeholder groups are outlined in Table 5.

**Table 5.** MoSCoW^a^ social features priorities for user-facing mobile app interface capabilities.

Description		Young people	Mental health researchers	PPIE^b^ leads	Included?	Reason for exclusion
**Social Features**						Resource limitations preclude the ongoing monitoring necessary to enable social features
	Ability for users to connect with one another, the public and other researchers	—^c^	Must Have	—	No	—
	Private messages	—	Should Have	—	No	—
	Area for users to share experiences, blogs, stories, etc	—	Should Have	—	No	—
	Ability to have conversations between researchers and contributors	—	Should Have	—	No	—
	Ability to share outside the platform	—	Should Have	—	No	—
	Linkable to social media outcomes	—	—	Should Have	No	—
	Area to share findings with the public	—	—	Should Have	No	—
	Monitored chatroom for personal interaction	Could Have	—	—	No	—
	Areas for more creative outputs	—	Could Have	—	No	—
	Input to write blog articles	—	—	Could Have	No	—
	Ability to connect with people with similar interests	—	—	Could Have	No	—

a MoSCoW: must have, should have, could have, won’t have.

b PPIE: patient and public involvement and engagement.

c Not applicable.

#### Design Features

Participants emphasized that VoiceIn should have a visually appealing and easy-to-navigate design. Specific design feature priorities across stakeholder groups are outlined in Table 6.

**Table 6.** MoSCoW^a^ design features priorities for user-facing mobile app interface capabilities.

Description of design features	Young people	Mental health researchers	PPIE^b^ leads	Included?	Reason for exclusion
Customizable notifications	Must Have	—^c^	—	No	Considered for future iterations
Easy to navigate back to the home screen	—	Must Have	—	Yes	—
Academic diary	Should Have	Should Have	—	No	Falls beyond the scope of the app
Video guide on how to use app	—	—	Could Have	No	Resource limitations preclude the creation of a video guide

a MoSCoW: must have, should have, could have, won’t have.

b PPIE: patient and public involvement and engagement.

c Not applicable.

#### Mental Health Researchers’ Priorities for App Functionality

Mental health researchers were also asked to rate their priorities for VoiceIn app functionality. The priorities are outlined in Table 7.

**Table 7.** MoSCoW^a^ priorities for app functionality.

Feature	Mental health researchers	Included in the app?
Users can sign up on a mobile app	Must Have	Yes
Users have a password	Must Have	Yes
User selects interests from pre-populated list	Must Have	Yes
Users can view research projects added by research on the web interface in the app	Must Have	Yes
Easy to navigate back to the home screen	Must Have	Yes
Users can “like” or “dislike” project cards to express interest	Must Have	Yes
Users can “like” a project to see more details	Must Have	Yes
Users who have “liked” a project and have chosen to contribute can respond to researcher polls & comments	Must Have	Yes
Users can view projects they have fed back on	Must have	Yes
Users contribute using both free text and polls	Must Have	Yes
Users can sign up using a handle of their choice	Should Have	No
User confirms birth year to verify age group	Should Have	Yes
Users can view progress on projects they have fed back on	Should Have	Yes
Users that have “liked” a project and have chosen to contribute can choose if they want to receive updates on the project.	Should Have	Yes
Users have an area where they can change preferences (topic interests)	Should Have	Yes
Users can choose to contribute anonymously (feedback does not have a user ID visible, but feedback can be seen by other users)	Could Have	Yes
Users can choose to contribute publicly (feedback has user ID visible)	Could Have	Not Yet
Users can change privacy preferences for visibility	Could Have	Not Yet
Users that have “liked” a project and chosen to contribute can see other responses to researcher polls	Could Have	Not Yet
Users that have “liked” a project can save it for later	Could Have	Yes
Users that have “disliked” a project have it removed from their card stack and do not receive notifications about this project again	Could Have	Yes
Users that have “liked” a project then can choose not to contribute to the project; project will be removed from their card stack and they will not receive notifications	Could Have	Yes
Sign up requires identifiable information	Won’t Have	No
Users can choose to contribute directly to the researcher (only researcher can see feedback; may or may not have user ID)	Won’t Have	No

a MoSCoW: must have, should have, could have, won’t have.

### Phase 2 Co-Design Workshops

The phase 2 workshops focused on asking participants to try out aspects of the tool and discuss the refined design prototypes. The prototypes showed how the mobile app and web-based researcher platform would look and function. In the workshops, participants were offered the opportunity to create a mock VoiceIn project, provide feedback on the ease of use of the VoiceIn platform, and identify areas of improvement.

Results of written feedback on the ease of use and areas of improvement from the usability testing workshops are presented in Table 8.

**Table 8.** Summary of usability feedback across stakeholder groups.

Feedback category	Young people	Mental health researchers	Changes incorporated
Ease of use	Easy to understand	First cohort rated the website as 5/5 in terms of ease of use	—^a^
Ease of use	Easy to engage with	Second cohort rated the website as 4.9/5 in terms of ease of use	—
Features that were good about the app or website	Use of videos in project descriptions	Clean and tidy design	—
Features that were good about the app or website	Choice about whether to contribute to each project	Straightforward to use	—
Features that were good about the app or website	“Quick and fun” to sort through project cards	Guides users through the process of setting up PPIE^b^ events	—
Features that were good about the app or website	Project updates section	—	—
Features that were good about the app or website	Ability to save projects for later	—	—
Features that were missing from the app or website or could be improved	Include description along with title on the project cards	Funder details should be included	Description incorporated
Features that were missing from the app or website or could be improved	—	Project start or end dates	Project start or end dates added to project details page
Features that were missing from the app or website or could be improved	—	Inclusion and exclusion criteria as separate boxes	Can be defined on per project basis by the researcher
Features that were missing from the app or website or could be improved	—	Distress and debrief section	Support page added signposting users to mental health support
Features that were missing from the app or website or could be improved	—	Use service-user defined categories like “self-harm” and “low mood” instead of diagnostic labels like “depression” to categorize studies	Categories expanded to include nondiagnostic categories like “hearing voices” and “loneliness”
Features that were missing from the app or website or could be improved	—	Add “potential impact of project“ section	Researchers have option to include this as part of the project description
Features that were missing from the app or website or could be improved	—	Section to outline expectation about time commitment and necessary skills	—
Features that were missing from the app or website or could be improved	—	More nuanced age range for study participants	—
Features that were missing from the app or website or could be improved	—	“Home“ button that returns users to home screen	“Home” button added
Features that were missing from the app or website or could be improved	—	More colorful design	—
Additional suggestions	—	Flexible project details page so it is fit-for-purpose for each project	Flexible project description section which enables the use of additional headings, photos, and videos

a Not applicable.

b PPIE: patient and public involvement and engagement.

**Figure 5.**
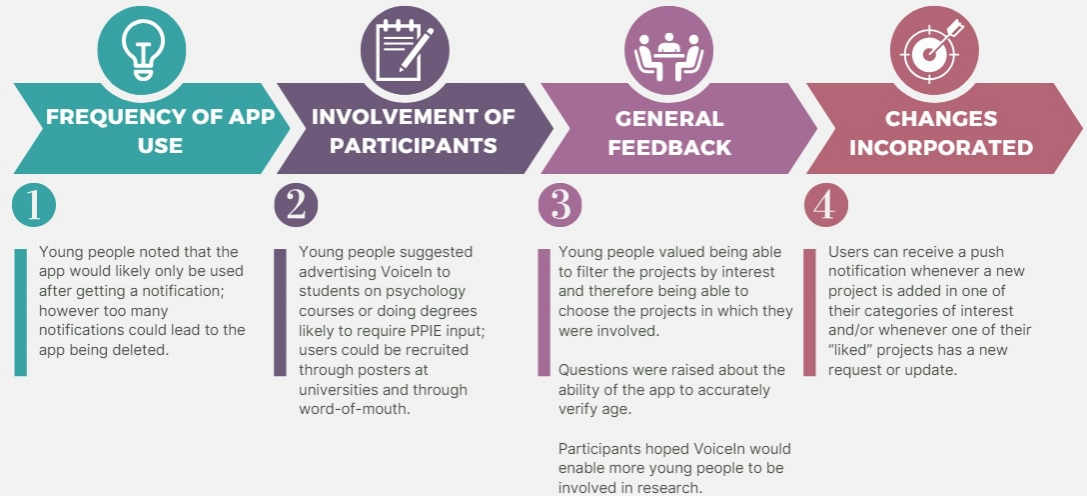
Summary of young people’s feedback on VoiceIn and its potential use. PPIE: patient and public involvement and engagement.

**Figure 6.**
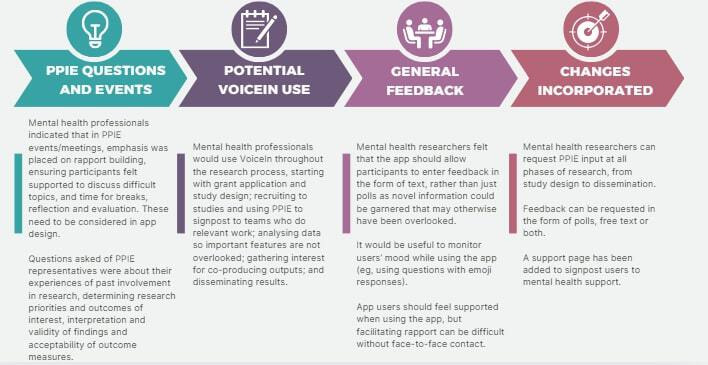
Summary of mental health researchers’ feedback on VoiceIn and its potential use. PPIE: patient and public involvement and engagement.

**Figure 7.**
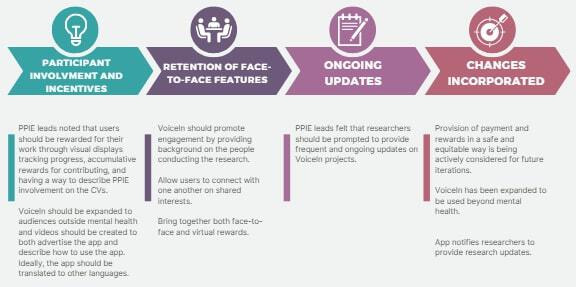
Summary of PPIE leads’ feedback about VoiceIn and its potential use. CV: curriculum vitae; PPIE: patient and public involvement and engagement.

In the phase 2 workshops, participants were also invited to give general feedback about the app and its potential use. A summary of this discussion and the changes incorporated in subsequent iterations of the app are described in Figures 5-7.

### Final VoiceIn Iteration

#### VoiceIn Mobile App

**Figure 8.**
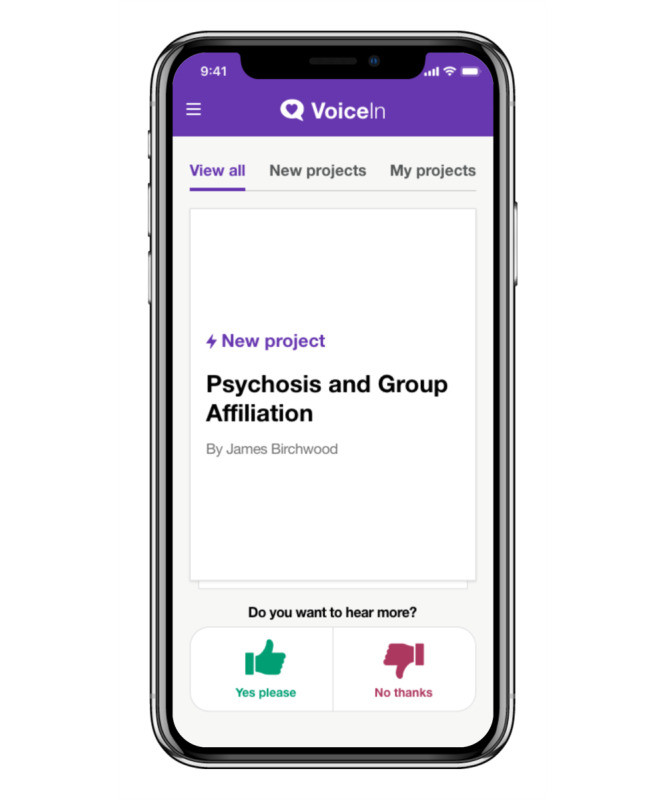
VoiceIn home screen where participants can view all projects.

The final iteration of the VoiceIn mobile app allows users to choose their research interests from a range of categories (eg, mental health and public health). Users are then brought to the home screen, in which relevant projects appear and users choose whether they are interested in finding out more about the project. When a user indicates they are interested in a project, they are shown the project description and asked if they would like to provide feedback on the project. Users then have the ability to answer questions (both poll and free text) and view project updates. In instances where feedback was provided via a poll, users will have the opportunity to see the distribution of responses. Interests and privacy settings can be changed by the user at any point. Sample images of the VoiceIn app are displayed in Figures8^11^.

**Figure 9.**
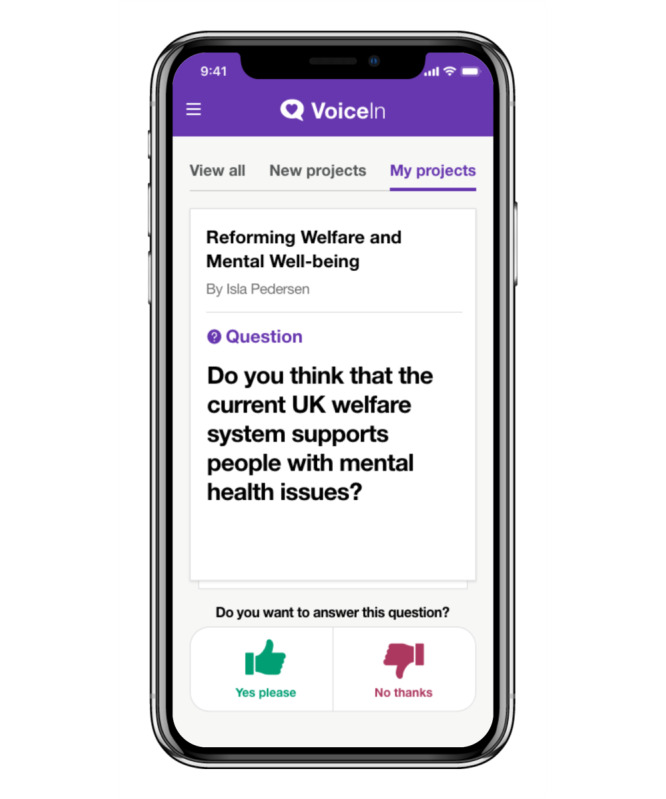
“My projects” page.

**Figure 10.**
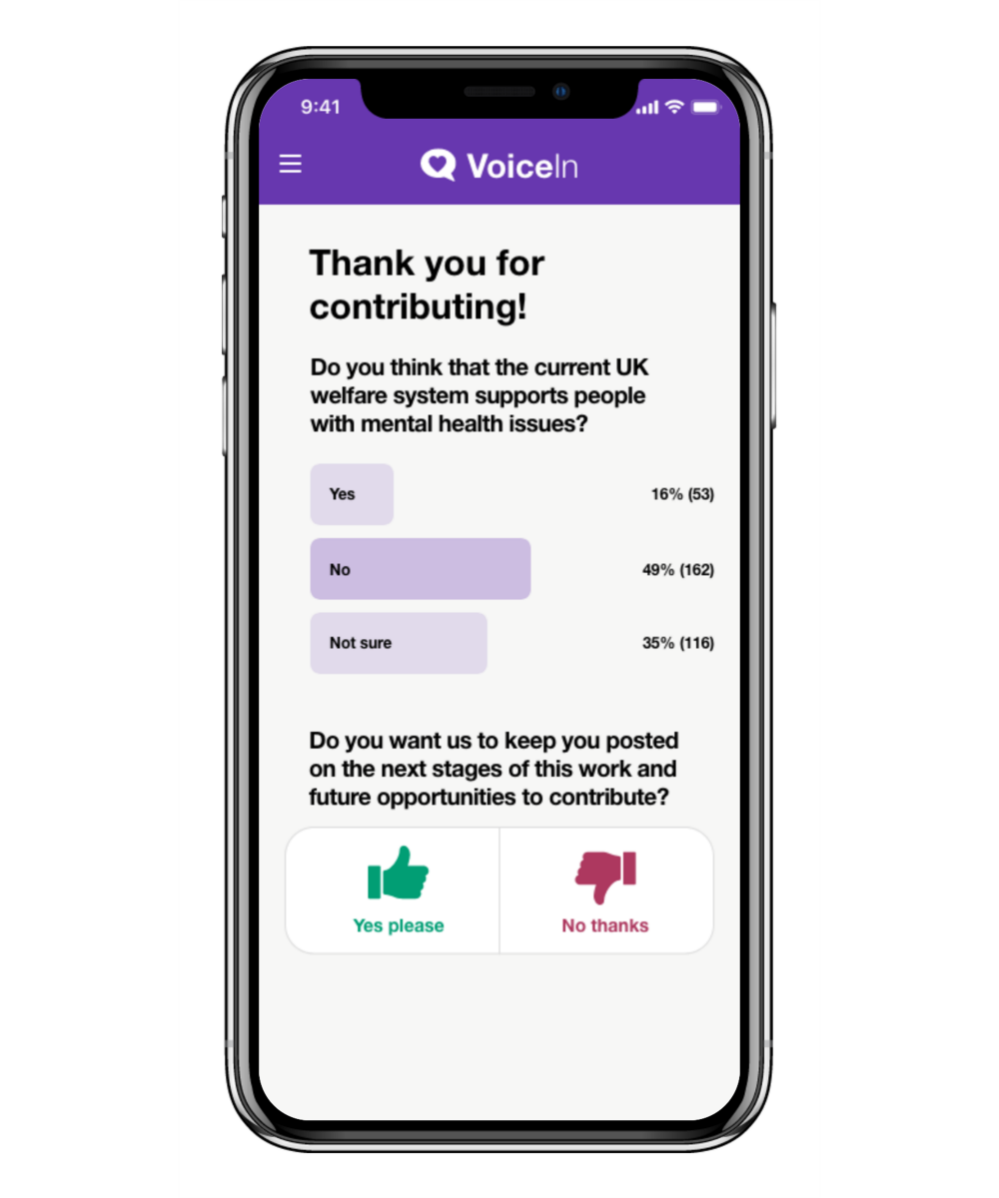
Sample distribution of responses to a poll.

**Figure 11.**
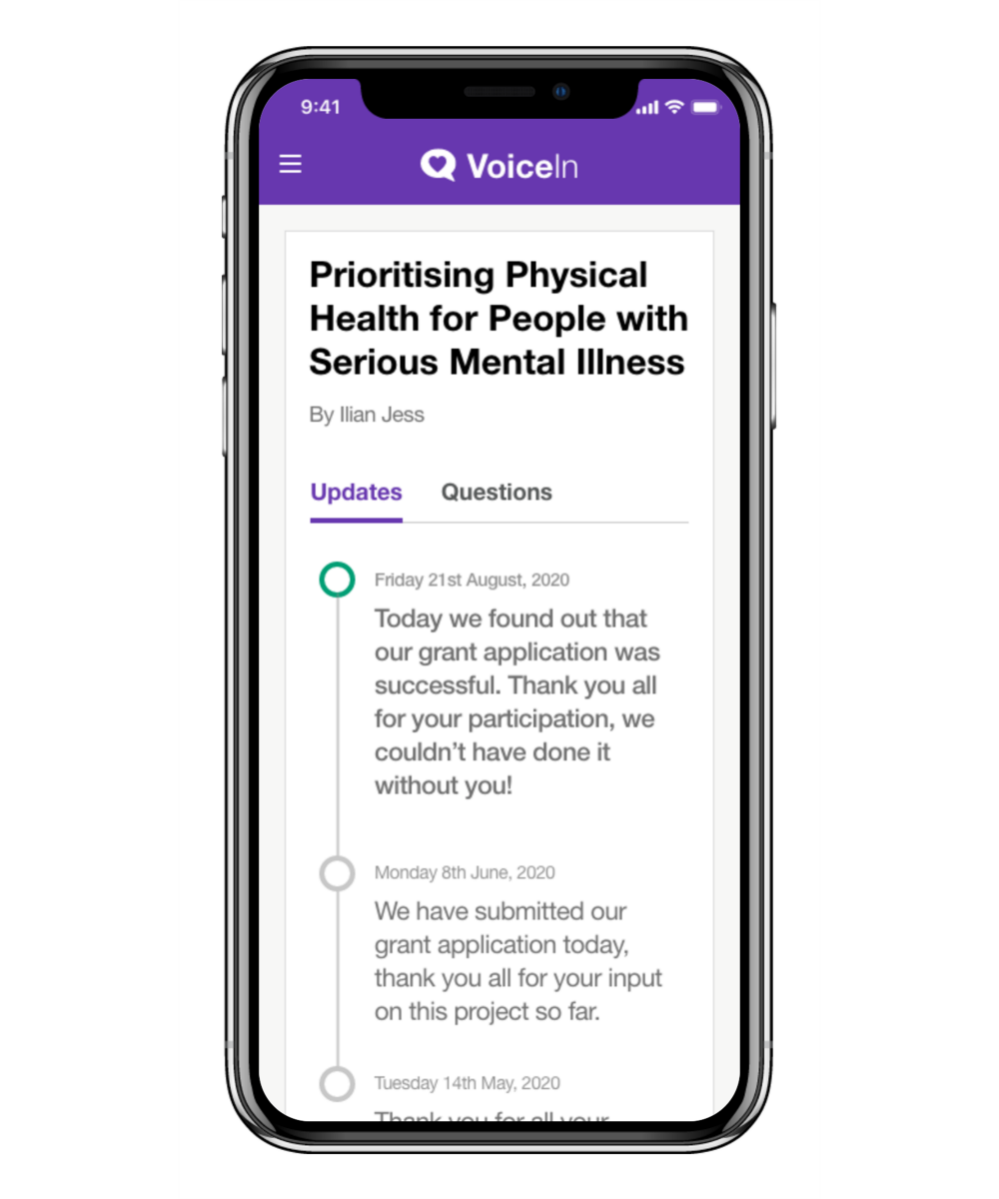
Sample “project updates” page.

#### Researcher-Facing Web Platform

**Figure 12.**
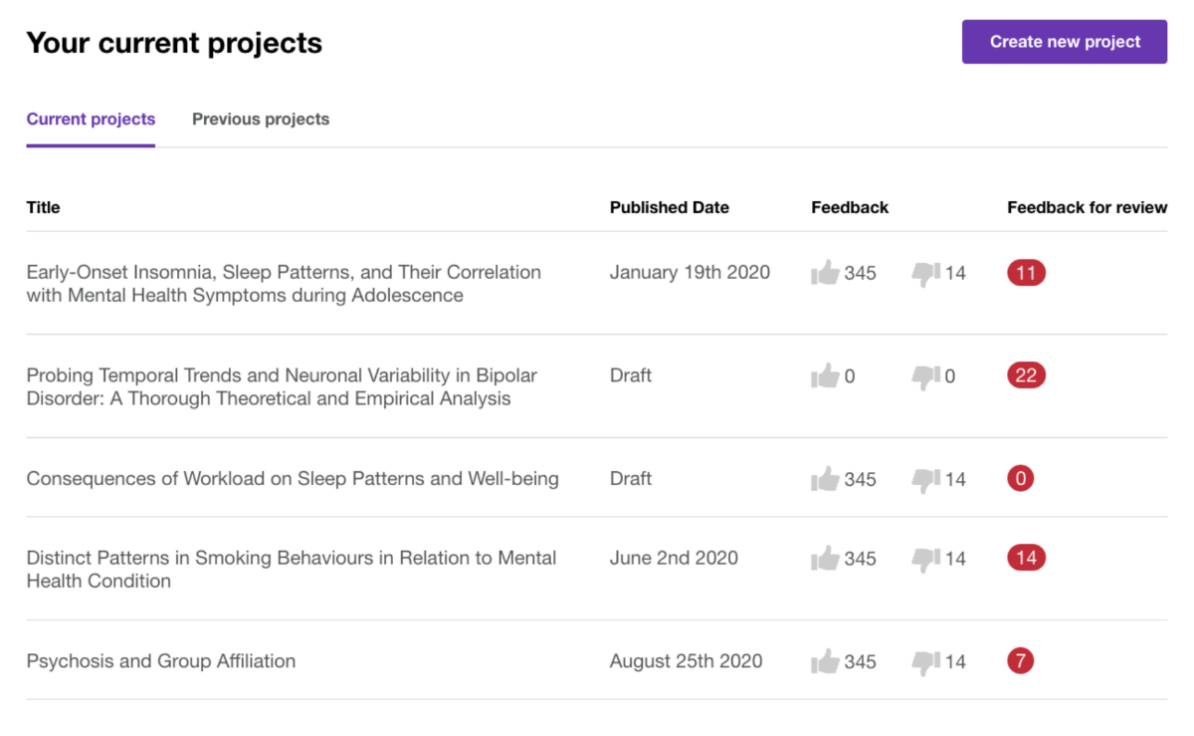
Sample “current projects” page for researchers.

VoiceIn was designed to enable researchers to have flexibility with how they present and elicit feedback for their projects. The home screen displays all of a researcher’s active projects and the amount of feedback received per project. When creating a new project, researchers insert a project title and indicate the intended audience (eg, age and interest), start and end dates, and terms of reference for the project. Project details can be displayed in the form of text, images, or videos; feedback can be requested as a poll or free text; and additional study documents can be uploaded. Once responses have been collected, researchers can view the demographic breakdown of participants, as well as see the poll and free text responses. Throughout the project, researchers can ask new questions, provide project updates, and review feedback to be displayed publicly on the app. Sample images of the web interface are displayed in Figures12  13.

**Figure 13.**
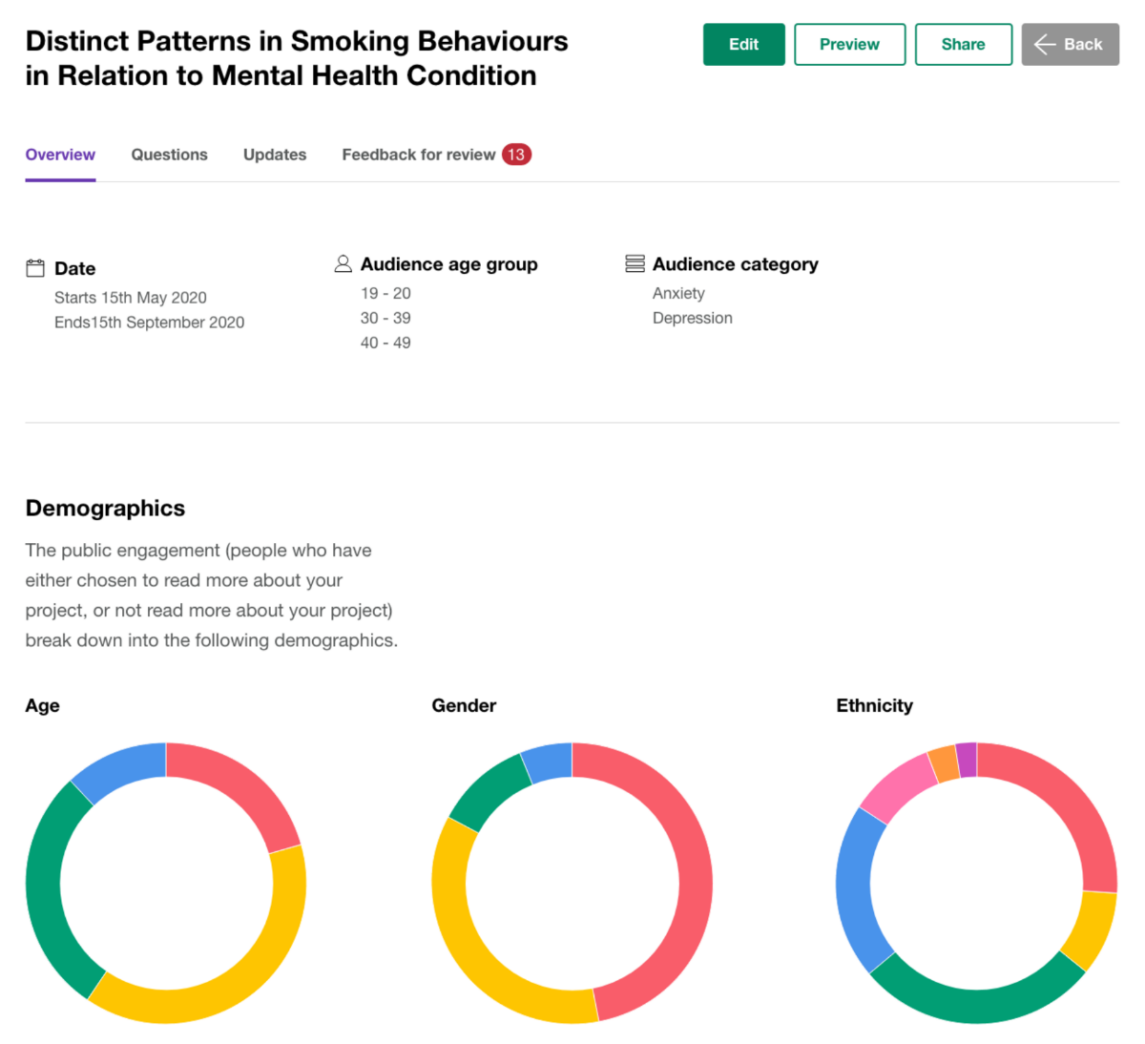
Sample demographics breakdown.

## Discussion

### Principal Findings

This paper outlines the process of co-designing the first digital tool to our knowledge that has been developed to enable real-time digital PPIE in mental health research. It likewise details the features that were identified as most important for young people, mental health professionals, and PPIE leads alike. Across stakeholder groups, participants highlighted that the VoiceIn app needed to be accessible, flexible, and easy to fit into the lives of young people. The key features that were included in the app were a flexible description of the research project, which can include the use of images and videos; numerous ways of engaging with the app and providing feedback on projects, including polls and free-text response options; and progress updates so that users can see how their input impacted the project and hear about how the research is making a difference in the world.

Co-designing the app with key stakeholders enabled the team to explore unique perspectives on developing a purely digital form of PPIE. The co-design process supported the development of an app that responded to the needs of our target user groups. It identified key features of the platform that were required for a usable and useful experience; supported feedback on the design and layout of the mobile app and web-based platform; established how a digital form could both supplant and supplement face-to-face PPIE activities; and highlighted the necessity of face-to-face modes of PPIE for in-depth discussion and exploration. It identified the potential for a mobile app that is accessible 24/7, and without geographical or spatial boundaries, to reach audiences often not represented in face-to-face activities.

The use of Agile software development methods enabled iterative versions of the app to be taken to app co-design workshops. This allowed end users to see the development of the app and the integration of features and changes that had been requested at previous workshops. Working iteratively with the software and research team, the Agile approach supported an ongoing process of iterative refinements and integration of feedback from the groups.

Time and budget inevitably constrained the breadth and depth of requirements that the software team was able to implement. Difficult choices on which features and functions to include in the app had to be made to ensure that the project met key milestones for delivery. Moreover, the co-design workshops, which were run by videoconference, encountered some of the problems that the VoiceIn app is designed to tackle. The co-design workshops required a reasonably long time commitment from people (90 mins); PPIE was conducted in a shared group space, which we recognize some may find challenging; and the research team struggled in some instances to recruit participants from diverse backgrounds. In the future, traditional modes of requesting co-design input could be supplemented by using the VoiceIn app to request feedback on itself by polling users.

Some challenges described during the co-design workshops were difficult to fully resolve and may require further iteration after live deployment. The challenge of remuneration of public users or contributors was difficult to resolve. Unlike traditional PPIE where fixed fees are usually paid for attending PPIE sessions, the groups suggested that payment for short contributions via the app was difficult to cost and to administer. Future versions of VoiceIn will consider remuneration in light of the philosophies of both PPIE, which promotes deep involvement in research, and citizen science, which may privilege rapid feedback [31]. VoiceIn will be more easily harmonized with rapid feedback models, many of which rely on voluntary involvement and nonmonetary incentives for involvement.

### Next Steps

The potential of the app to support PPIE for mental health research has not yet been tested. The next phase of the project is to test the app. User needs will continue to be identified as the app is rolled out and piloted in the United Kingdom. The co-design and stakeholder involvement will continue to capture user needs after the app is in real-world use. A benefit of VoiceIn is that we can use the app to capture feedback in real-time, as well as track data analytics on how people use the app. Real-time feedback and analytics allow researchers to respond to problems or improvements identified. At the time of writing, we believe this is a world-first platform for digital PPIE; the potential for VoiceIn to reach a wider audience for PPIE than more traditional or face-to-face methods and to develop a learning health system for PPIE remains to be tested.

Our overarching goal is for VoiceIn to support PPIE activities for anyone, anywhere who would find it helpful. Our initial aim is for the platform to be widely used across the United Kingdom to support PPIE in health research broadly. Ultimately, we aim to scale the platform to support global research studies.

### Conclusion

VoiceIn can support PPIE by providing an easy-to-use, digital interface that enables public contributors to share ideas and feedback on research projects from the comfort of their smartphone, at times that are convenient to them. For researchers, VoiceIn offers a new way to engage public contributors who otherwise may be difficult to access or who may not like to participate in face-to-face PPIE activities.

VoiceIn is not a solution for all PPIE needs and activities. There remains an important need for traditional and face-to-face ways of involving and engaging public contributors that can support sustained and meaningful conversations and more detailed exploration and discussion of ideas. Digital inequalities, such as lack of access to technology and lack of skills or confidence to use digital technology, also mean that VoiceIn is not an accessible solution for all. However, for VoiceIn’s target audience of young people aged 16‐25 years, providing an option to contribute to research via their smartphone was seen as convenient and appealing.

While VoiceIn was initially developed for mental health research, it has now been adapted to accommodate health research more broadly. The next phase of the project involves releasing the VoiceIn app onto the public app marketplace and enrolling research projects onto the platform. Our co-design work indicates that there is both a need and an appetite for this digital mode for PPIE. However, it remains to be seen if VoiceIn will be widely used by researchers and public contributors. The potential for VoiceIn to revolutionize PPIE by enabling individuals and groups who may have previously felt excluded from face-to-face or traditional PPIE activities and by supporting researchers to reach a wider public audience now needs to be tested.

## Abbreviations

MoSCoW: must have, should have, could have, won’t have
PPIE: Patient and public involvement and engagement
